# Air Pollution and Symptoms of Depression in Elderly Adults

**DOI:** 10.1289/ehp.1104100

**Published:** 2012-04-18

**Authors:** Youn-Hee Lim, Ho Kim, Jin Hee Kim, Sanghyuk Bae, Hye Yin Park, Yun-Chul Hong

**Affiliations:** 1 Department of Epidemiology and Biostatistics, and; 2 Institute of Health and Environment, Graduate School of Public Health, Seoul National University, Seoul, Republic of Korea; 3 Institute of Environmental Medicine, Seoul National University Medical Research Center, Seoul, Republic of Korea; 4 Environmental Health Center, and; 5 Department of Preventive Medicine, Seoul National University College of Medicine, Seoul, Republic of Korea; 6 Institute of Medical Research, Seoul National University Bundang Hospital, Seongnam, Republic of Korea

**Keywords:** air pollution, depressive symptoms, elderly, factor analysis, panel study

## Abstract

Background: Although the effect of air pollution on various diseases has been extensively investigated, few studies have examined its effect on depression.

Objectives: We investigated the effect of air pollution on symptoms of depression in an elderly population.

Methods: We enrolled 537 participants in the study who regularly visited a community center for the elderly located in Seoul, Korea. The Korean version of the Geriatric Depression Scale-Short Form (SGDS-K) was used to evaluate depressive symptomatology during a 3-year follow-up study. We associated ambient air pollutants with SGDS-K results using generalized estimating equations (GEE). We also conducted a factor analysis with items on the SGDS-K to determine which symptoms were associated with air pollution.

Results: SGDS-K scores were positively associated with interquartile range (IQR) increases in the 3-day moving average concentration of particulate matter with an aerodynamic diameter ≤ 10 μm (PM_10_) [17.0% increase in SGDS-K score, 95% confidence interval (CI): 4.9%, 30.5%], the 0–7 day moving average of nitrogen dioxide [NO_2_; 32.8% (95% CI: 12.6%, 56.6%)], and the 3-day moving average of ozone [O_3_; 43.7% (95% CI: 11.5%, 85.2%)]. For these three pollutants, factor analysis showed that air pollution was more strongly associated with emotional symptoms such as feeling happy and satisfied than with somatic or affective symptoms.

Conclusions: Our study suggests that increases in PM_10_, NO_2_, and O_3_ may increase depressive symptoms among the elderly. Of the symptoms evaluated, ambient air pollution was most strongly associated with emotional symptoms.

Depression is one of the common mental health problems experienced by the elderly and has been found to lead to increased mortality and suicide in this age group (Blazer et al. 2001; Waern et al. 2003). Known risk factors for depression among the elderly are alcohol and substance abuse, sleep disturbance, bereavement, medical conditions, family history, and being female (Cole and Dendukuri 2003; Mulsant and Ganguli 1999). Hypotension and a low lipid level also have been found to be associated with depressive symptoms among the elderly and women, respectively (Horsten et al. 1997; Kim et al. 2010).

Plausible biological mechanisms of depression include reactivity to exogenous stressors; alterations of neurohumoral, immune, and autonomic regulation; dysfunction of neurotransmitter systems; and oxidative stress (Grippo 2009; Ng et al. 2008). Because depletion of dopamine in the central nervous system (CNS) is known to be an underlying pathophysiological mechanism of depression (Hasler 2010), air pollution could affect depressive moods by inducing dopaminergic neurotoxicity, possibly due to oxidative stress (Block et al. 2004). Research with cell cultures and experimental animals has provided evidence of neuropathological effects of exposure to particles (Block et al. 2004; Campbell et al. 2005; Veronesi et al. 2005). However, only a few studies have evaluated the association between air pollution and depressive symptoms in humans. In Canada, researchers reported short-term effects of air pollution on emergency department visits because of depression and suicide attempts (Szyszkowicz 2007a; Szyszkowicz et al. 2009b, 2010). Their time-series analyses focused on the diagnosis of depression resulting from severe pathophysiological alterations in the CNS, and their results suggest that air pollution may have aggravated symptoms of depression among a pool of patients in the community who were suffering from depression. However, Bullinger (1989) has proposed that short-term exposure to air pollution more likely affects daily mood rather than causes major depressive illnesses. Although studies have found that major (Valvanne et al. 1996) or minor (Lyness et al. 2006) depression is common among the elderly, who may also be most susceptible to adverse health effects of air pollution (Pope 2000), no studies have examined changes in depressive symptoms associated with short-term exposure to air pollution among the elderly.

Our study was based on an *a priori* hypothesis that air pollution may affect depressive symptoms, because air pollution is known to induce oxidative stress, a potential cause of depression. Hence, we examined the effect of air pollution on symptoms of depression among the elderly in Seoul, Korea, from 2008 to 2010, by evaluating depressive symptom test scores, as well as individual symptoms that contributed to the overall score.

## Materials and Methods

*Population.* This study evaluated data from 560 participants who regularly visited a community welfare center for the elderly located in Seongbuk-Gu. This region is one of 25 districts in Seoul, Korea, located in the northern midsection of the city where approximately 460,000 residents reside in an area approximately 25 km^2^.

We performed the first follow-up of this 3-year study from August 2008 to December 2008, the second from April to October 2009, and the third from March to August 2010. We excluded persons diagnosed with dementia or Alzheimer’s disease (*n* = 23) because of concerns regarding compliance and reliability. During the 3-year follow-up period, 166 participants visited the welfare center only once, 184 visited twice, and 187 visited three times. Baseline questionnaires were administered by interviewers and were used to record demographic and behavioral characteristics, a self-reported history of physician-diagnosed chronic diseases, at least 1-year of medication use for the reported chronic diseases, and dietary intake of selected items, such as meat, fish, and vegetables. At each visit, participants underwent a physical examination and answered questions on the depression symptom questionnaire. Weight and height were measured, and body mass index (BMI) was calculated (kilograms per meter squared). Mean systolic blood pressure (SBP; millimeter of mercury) and diastolic blood pressure (DBP; millimeter of mercury) were calculated from two measurements taken at intervals of ≤ 5 min. Fasting blood and urine samples were drawn between 0900 and 1200 hours on the same day. We obtained written informed consent from all participants, and the institutional review board of Seoul National University Hospital reviewed and approved the study protocol.

*Measure of depressive symptoms.* To evaluate depressive symptoms during the previous 1 week, trained staff conducted face-to-face interviews using the Korean version of the Geriatric Depression Scale-Short Form (SGDS-K) (Bae and Cho 2004). The SGDS-K consists of 15 items (5 positive and 10 negative feelings for the previous week) each coded as either negative (1) or positive (0), with total scores ranging from 0 to 15. We inverted responses to questions about positive feelings so that higher summed scores would indicate greater depression severity. To assess the reliability of the SGDS-K responses, we conducted a test-retest comparison among 30 randomly selected participants who completed two study visits 1 week apart (the first interview was conducted from 8 March to 10 March 2011 and the second from 15 March to 17 March 2011).

*Environmental variables.* We obtained air pollution data from the Research Institute of Public Health and Environment [Ministry of Environment (MOE) 2009, 2010, 2011]. For Seongbuk-Gu residents (90% of the study population), one monitor, which was centrally located in Seongbuk-Gu, was used as a proxy for individual exposure to ambient pollutants. For other residents, the nearest monitoring site to their residential address was used as a measure of air pollution concentrations.

Particulate matter with an aerodynamic diameter ≤ 10 µm (PM_10_) was measured based on the radiometric principle of beta attenuation (Sensor FH62C14; Thermo Fisher Scientific Inc., Franklin, MA, USA). Carbon monoxide (CO) was measured using an infrared sensor (EC9830B CO Gas Analyzer; ECOTECH, Victoria, Australia), sulfur dioxide (SO_2_) by a fluorescent method (EC9850B SO2 Gas Analyzer; ECOTECH), nitrogen dioxide (NO_2_) by a chemiluminescent method (EC9841B NO2 analyzer; ECOTECH), and ozone (O_3_) by a ultraviolet photometric method (EC9810B; ECOTECH). All monitors were located on the rooftops of municipal buildings, and measurements were made every 5 min. For each site, we computed daily mean values of PM_10_, SO_2_, and NO_2_, and daily maximum values of CO and O_3_ between 0900 and 1800 hours. Vehicle emissions are responsible for a substantial share of air pollution concentrations in this area, as in other areas in Seoul (Kim and Guldmann 2011). We also collected temperature and rainfall values from the Korea Meteorological Administration (2012) to adjust for these environmental confounders in the model (Keatinge and Donaldson 2001).

*Statistical analysis.* We performed multivariate analysis using generalized estimating equations (GEE) to estimate the effects of air pollution on depression. The multivariate analysis also accounted for the correlated structure of the data due to repeated measures over time. We modeled scores of SGDS-K as a Poisson distribution that assumed compound symmetry and that each item of symptomatology was a binomial distribution. We expressed estimated effects of an interquartile increase in the concentration of each individual air pollutant as the percent change in total SGDS-K scores and as odds ratios (ORs) for negative responses for individual symptom items. In all models, we included factors that we hypothesized *a priori* could potentially confound the relationship between air pollution and depression. Time-independent factors measured at baseline included age (years), sex (male or female), number of years of school (< or ≥ 6 years), BMI, alcohol consumption at least once a month for 10 years or longer (yes or no), and moderate physical activity at least once a week (yes or no). Time-varying factors included creatinine-adjusted urinary cotinine level as a proxy of exposure to either passive or active smoking, mean temperature and rainfall level (0 = no rain, 1 = below mean, 2 = above mean level of daily total rain) on the day of each visit, visit number (1st, 2nd, or 3rd visit), and day of the week (Monday through Sunday). We also controlled for serum triglyceride (milligrams per deciliter) and SBP measured at each visit, which have been previously associated with depressive symptoms (Horsten et al. 1997; Kim et al. 2010).

We estimated delayed effects of air pollution on depressive symptoms using the following moving average lag structures: 0 day (pollutants measured on the day of the study visit), 0–2 days (the same day and the previous 2 days), 0–5 days, 0–7 days, 0–14 days, 0–21 days, and 0–28 days. When reporting the results, we focused on the lag structure that provided the best fit to the data based on the quasi-likelihood information criterion (QIC) (Pan 2001). Alpha level was set at 95%, and statistical significance was *p* < 0.05.

To investigate whether a history of cardiovascular disease (CVD) may modify associations between air pollutants and symptoms of depression, we stratified participants into two categories (yes or no) based on any CVD-related history, including hypertension, myocardial infarction (MI), hyperlipidemia, or stroke and examined effect modification by each individual CVD except stroke, which was reported by only 2% of our study population.

We estimated the effects of air pollution on each depressive symptomatology item as a dichotomous outcome using the lag structure that best fit the lowest QIC value, as previously described, in a GEE model with a binomial distribution adjusted for confounders. In addition, we performed a factor analysis of principal components with varimax rotation for the 15 items of SGDS-K to determine which items appeared to contribute to a common factor. For each factor, we generated composite scores calculated as the loading value–weighted sum of binary scores (0 or 1) of each individual item that contributed to the factor. We expressed estimated effects of an interquartile increase in the concentration of each individual air pollutant as the percent change in composite scores.

Numbers of repeated measures (study visits) varied among participants, which may lead to selection bias if numbers of visits are not random (Rubin 1976). Analysis was conducted after weighting follow-up observations by the inverse probability of attaining a follow-up response (Robins et al. 1995). For participants with more than one visit, we conducted logistic regression to predict the probability of follow-up (follow-up = 1, missing = 0) with covariates of the previous measurements including age, sex, BMI, number of years of school, blood pressure, season, and temperature. We gave a weight of 1 to the first observation for each participant and more weight (the inverse of predicted probability of having a follow-up response) to the observations that were more likely missing (McCracken et al. 2010).

For the sensitivity analysis, we constructed single- and two-pollutant models based on five pollutants—PM_10_, NO_2_, O_3_, CO, and SO_2_—and observed any attenuation of a single-pollutant effect by adjusting for another pollutant in the model.

## Results

Among the 537 participants at the first (baseline) study visit, the average age was 71 years, BMI was 25 kg/m^2^, and creatinine-adjusted urine cotinine as a proxy of exposure to either passive or active smoking was 255 mg/g (Table 1). The percentage of female participants was 74%. The proportion of individuals who reported regular alcohol consumption at least once a month for ≥ 10 years was 23%, and 63% of participants reported moderate exercise at least once a week. Hypertension was the most common preexisting disease (51%) followed by diabetes (18%) (Table 1).

**Table 1:** Description of the study population at the first study visit (n = 537).

Variable	Mean ± SD (median, range)
Age (year)	71 ± 5 (70, 60–87)
Female [n (%)]	396 (74)
BMI (kg/m2)	25 ± 3 (25, 15–36)
Creatinine-adjusted urine cotinine (mg/g)	255 ± 1,256 (2, 0–11,866)
SBP (mmHg)	131 ± 17 (130, 90–190)
Serum triglyceride (mg/dL)	141 ± 101 (118, 30–1,331)
Regular alcohol drinking [n (%)]	118 (23)
Regular exercise [n (%)]	331 (63)
≤ 6 years of school [n (%)]	309 (58)
Monthly income < 500 US$ [n (%)]	440 (84)
Insurance benefit from community or government [n (%)]	201 (39)
Living without spouse or partner [n (%)]	214 (41)
Stroke [n (%)]	9 (2)
Hypertension [n (%)]	274 (51)
MI [n (%)]	35 (7)
Hyperlipidemia [n (%)]	56 (10)
COPD [n (%)]	3 (1)
Asthma [n (%)]	8 (1)
Depression [n (%)]	6 (1)
Diabetes [n (%)]	83 (18)
COPD, chronic obstructive pulmonary disease.	

The average SGDS-K score out of a possible 15 was 3.6 at the first visit, 2.4 at the second visit, and 1.1 at the third visit (Table 2). The proportion of participants who responded negatively to each SGDS-K item was highest at the first visit. In the Supplemental Material, Table S1 (http://dx.doi.org/10.1289/ehp.1104100), we show correlations among SGDS-K items, factor loading values from a factor analysis, and reliability coefficients for the test-retest analysis. For the factor loading values, the first factor consisted of six items (1, 5, 6, 7, 8, and 11), which may be interpreted as a dimension of emotional symptoms. The second factor was related to somatic symptoms and covered three items (9, 10, and 13). The third factor included six items (2, 3, 4, 12, 14, and 15) related to affective symptoms. The results of test-retest analysis at a 1-week interval showed a high correlation (Pearson *r* = 0.92) of SGDS-K scores (see Supplemental Material, Table S1).

**Table 2:** Depressive symptom scoresa [mean ± SD (median, range)].

Variable	1st follow-up August–December 2008 (n = 383)	2nd follow-up April–December 2009 (n = 368)	3rd follow-up March–August 2010 (n = 344)
Total SGDS-K score		3.6 ± 3.3 (3, 0–15)		2.4 ± 3.3 (1, 0–14)		1.1 ± 2.3 (0, 0–14)
Factor 1 (emotional symptoms)						
1. Satisfied with life		73 (26)		77 (21)		79 (23)
5. In good spirits		63 (22)		60 (16)		14 (4)
6. Fear bad things		67 (24)		46 (13)		5 (1)
7. Happy most of the time		59 (21)		59 (16)		13 (4)
8. Often feel helpless		51 (18)		18 (5)		4 (1)
11. Wonderful to be alive		53 (19)		46 (13)		11 (3)
Composite score of factor 1		0.8 ± 1.0 (0.5, 0–3.8)		0.5 ± 1.0 (0, 0–3.8)		0.2 ± 0.6 (0, 0–3.8)
Factor 2 (somatic symptoms)						
9. Prefer to stay home		34 (12)		23 (6)		32 (9)
10. Problems with memory		76 (27)		34 (9)		54 (16)
13. Full of energy		85 (30)		85 (23)		37 (11)
Composite score of factor 2		0.4 ± 0.5 (0, 0–1.9)		0.2 ± 0.4 (0, 0–1.9)		0.2 ± 0.5 (0, 0–1.9)
Factor 3 (affective symptoms)						
2. Dropped activities/interests		129 (46)		146 (40)		70 (21)
3. Life is empty		69 (24)		45 (12)		6 (2)
4. Often get bored		64 (23)		77 (21)		25 (7)
12. Feel pretty worthless		61 (22)		74 (20)		10 (3)
14. Situation is hopeless		87 (31)		55 (15)		6 (2)
15. Others are better off		39 (14)		33 (9)		7 (2)
Composite score of factor 3		0.9 ± 1.0 (0.6, 0–3.5)		0.7 ± 1.0 (0, 0–3.5)		0.2 ± 0.5 (0, 0–3.5)
an (%) of negative responses of individual items.						

Table 3 shows characteristics of environmental factors in Seongbuk-Gu during the study period (August through December 2008, April through December 2009, and March through August 2010). The mean ± SD temperature was 17.3°C ± 8.1, PM_10_ concentration was 43.7 µg/m^3^ [interquartile range (IQR) = 24.2], SO_2_ concentration was 4.0 ppb (IQR = 2.3), NO_2_ concentration was 36.2 ppb (IQR = 5.0), maximum CO was 0.79 ppm (IQR = 4.0), and O_3_ was 48.1 ppb (IQR = 37.0) (Table 3). Pollutants, except O_3_, showed a high correlation with other pollutants in repeatedly measured data and follow-up data [see Supplemental Material, Table S2 (http://dx.doi.org/10.1289/ehp.1104100)]. Although PM_10_ and SO_2_ concentrations did not show distinct patterns depending on hours of exposure, NO_2_ and CO concentrations were slightly higher during the morning rush hour (0700–0900 hours) and midnight through early morning (2300–0100 hours) than in other hours of a 24-hr day [see Supplemental Material, Figure S1a–e]. During the daytime (1400–1700 hours), high O_3_ concentrations were measured and were positively correlated with other pollutants [see Supplemental Material, Figure S1(f)].

**Table 3:** Overall characteristics of environmental factors in Seongbuk-Gu, Korea (August–December 2008, April–December 2009, and March–August 2010).

Mean ± SD	Median	Range	IQR
Mean temperature (°C)		17.3 ± 8.1		19.4		–7.2–29.2		14.0
Rainfall (mm)		6.9 ± 17.8		0.5		0–113.5		6.8
Mean PM10 (µg/m3)		43.7 ± 23.7		40.8		7.4–151.3		24.2
Mean SO2 (ppb)		4 ± 2.2		3.5		1–17.5		2.3
Mean NO2 (ppb)		36.2 ± 12.1		34.8		9.8–77.3		15.0
Maximum CO (10 ppm)		7.9 ± 4.3		7.0		3–27		4.0
Maximum O3 (ppb)		48.1 ± 27		44.0		2–140		37.0

**Figure 1:**
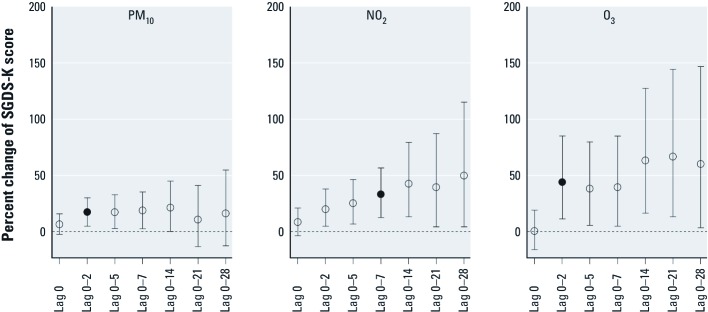
Percent change in SGDS-K scores per interquartile range (IQR) of air pollutants by increasing lag day. Solid circles indicate the best-selected lag days based on minimal Akaike Information Criterion. IQRs for PM_10_, NO_2_, and O_3_ were 24 µg/m^3^, 15 ppb, and 37 ppb, respectively. The model for each lag structure included the following variables: age, sex, number of years of school, BMI, alcohol consumption, regular exercise, creatinine-adjusted urine cotinine level, SBP, triglyceride, daily mean temperature, follow-up time, and day of the week.

Figure 1 displays the estimated percent change in SGDS-K scores per IQR increase in air pollutants at different lag days. The best fitting lag structures for PM_10_, NO_2_, and O_3_ for the overall depression score were lag 0–2 days, lag 0–7 days, and lag 0–2 days, respectively. IQR increases in PM_10_, NO_2_, and O_3_ for the best selected lag days were significantly associated with SGDS-K scores, with estimated increases of 17.0% [95% confidence interval (CI): 4.9%, 30.5%], 32.8% (95% CI: 12.6%, 56.6%), and 43.7% (95% CI: 11.5%, 85.2%), respectively. SO_2_ and CO were not significantly associated with SGDS-K scores at any moving average lag days within 4 weeks. IQR increases in SO_2_ apparently had a marginally protective effect at lag 0–21 days [–20.0% (95% CI: –36.6%, 0.9%)] and null effects at other lag days. Increases in CO showed positive associations at lag days 0–5, 0–7, and 0–14, and null associations at other lag days [see Supplemental Material, Figure S2 (http://dx.doi.org/10.1289/ehp.1104100)]. When we performed cross-sectional analysis using data from the first visit only, estimated effects of PM_10_ and NO_2_ on depressive symptoms were slightly attenuated but similar to associations based on the repeated measures analysis, although the magnitude of estimated effects of O_3_ were not statistically significant for any lag days and were considerably lower (see Supplemental Material, Figure S3).

**Figure 2:**
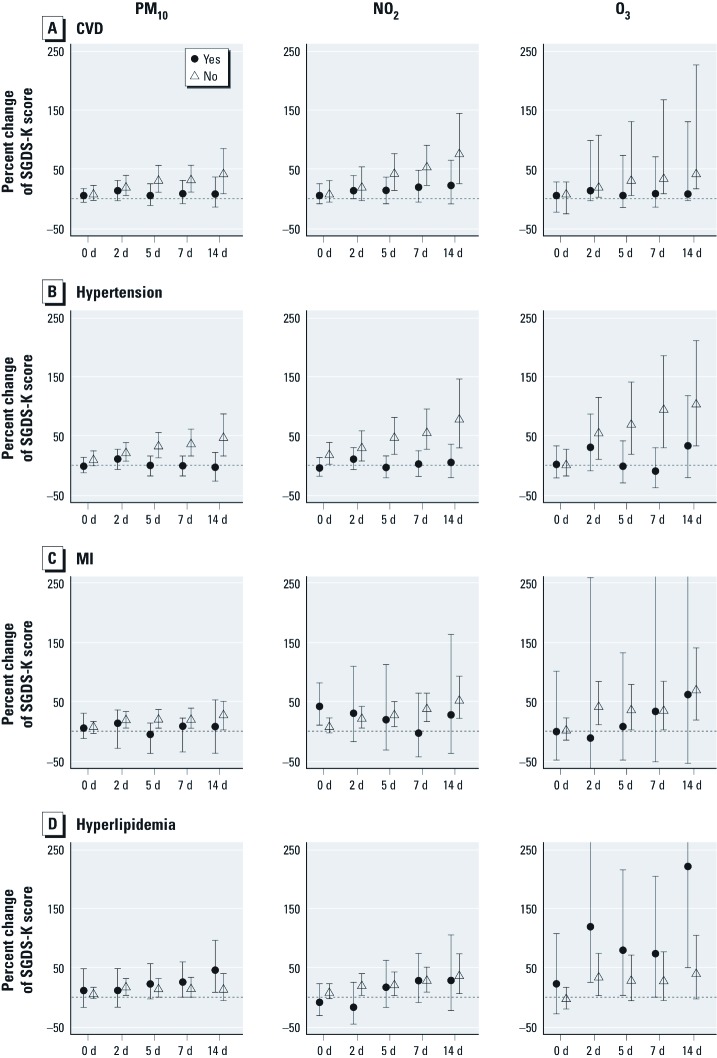
Percent change in SGDS-K scores by increasing lag days per IQR of air pollutants with data stratified by responses to a history of CVD [yes (solid circle)] vs. no (triangle)] (*A*), hypertension (*B*), MI (*C*), and hyperlipidemia (*D*). 0 d, concurrent exposure to air pollution; 2 d, moving average lag days from concurrent to the two previous days; 5 d, 0–5 lag days of moving average; 7 d, 0–7 lag days; and 14 d, 0–14 lag days. The model for each lag structure included the following variables: age, sex, number of years of school, BMI, alcohol consumption, regular exercise, creatinine-adjusted urine cotinine level, SBP, triglyceride, daily mean temperature, follow-up time, and day of the week.

We examined whether the association between depression and air pollution was modified by a history of CVD. Figure 2B suggests that participants without a history of hypertension may have been more susceptible to air pollution exposures than were those with a history of CVD. However, associations with O_3_ were stronger among participants with a history of hyperlipidemia than among those without this history. We observed little difference between participants with and without CVD (Figure 2A) or MI (Figure 2C).

Table 4 depicts the association of each individual item in the SGDS-K with IQR increases in PM_10_, NO_2_, and O_3_. All items in the first factor (emotional symptoms) showed significant associations with some or all of the three pollutants (PM_10_, NO_2_, and O_3_). ORs associated with responding negatively to items related to emotional symptoms (1, 5, 6, 7, 8, and 11) increased significantly with an IQR increase of NO_2_. Some of these items also showed a significant relationship with O_3_ and PM_10_. However, in the second and third factors that were related to somatic and affective symptoms, only “life is empty” was associated with three air pollutants, whereas “problems with memory,” “full of energy,” and “others are better off” were associated with one pollutant, and other items were not associated with any pollutants. Similarly, among the factor-specific composite scores (emotional, somatic, and affective symptoms), which were calculated as the loading value–weighted sum of the item responses within each factor structure, we observed that emotional symptom composite scores increased with increasing concentrations of PM_10_ [38.2% (95% CI: –3.6%, 98.1%)], NO_2_ [118.2% (95% CI: 37.9%, 245.3%)], and O_3_ [132.5% (95% CI: 32.0%, 309.3%)]; however, somatic and affective symptoms were associated with an increase of PM_10_, and nonsignificantly but positively associated with increasing NO_2_ and O_3_.

**Table 4:** Estimated effectsa of responding negatively to each SGDS-K itemb and composite scoresc per IQR of air pollutants (95% CI).

PM10 (IQR = 24 µg/m3)			NO2 (IQR = 15 ppb)			O3 (IQR = 37 ppb)		
Factor/Item	Estimates (95% CI)	Lag day	Estimates (95% CI)	Lag day	Estimates (95% CI)	Lag day
Factor 1 (emotional symptoms)												
1. Satisfied with life		1.01 (1.00, 1.03)*		0–5		1.04 (1.01, 1.07)*		0–7		1.03 (1.01, 1.06)*		0–28
5. In good spirits		1.01 (1.00, 1.02)		0–2		1.03 (1.01, 1.06)*		0–2		1.01 (0.98, 1.04)		0–28
6. Fear bad things		1.03 (1.01, 1.06)*		0–28		1.10 (1.03, 1.17)*		0–28		1.04 (1.01, 1.08)*		0–28
7. Happy most of the time		1.01 (1.00, 1.03)		0–2		1.11 (1.04, 1.18)*		0–28		1.03 (1.00, 1.06)		0–28
8. Often feel helpless		1.05 (1.02, 1.09)*		0–28		1.11 (1.04, 1.18)*		0–21		1.06 (1.02, 1.1)*		0–28
11. Wonderful to be alive		1.02 (1.00, 1.03)*		0–5		1.05 (1.02, 1.08)*		0–7		0.99 (0.96, 1.02)		0–28
Composite score of factor 1c		38.2% (–3.6, 98.1)**		0–28		118.2% (37.9, 245.3)*		0–28		132.5% (32.0, 309.3)*		0–28
Factor 2 (somatic symptoms)												
9. Prefer to stay home		1.02 (0.99, 1.05)		0–14		1.02 (0.96, 1.09)		0–14		1.03 (1.00, 1.06)		0–28
10. Problems with memory		1.04 (1.02, 1.06)		0–14		1.07 (1.02, 1.12)*		0–14		1.01 (0.99, 1.04)		0–28
13. Full of energy		1.02 (1.00, 1.03)*		0–2		1.03 (1.00, 1.05)		0–5		1.00 (0.98, 1.03)		0–28
Composite score of factor 2c		38.9% (0.7, 91.7)*		0–21		36% (–8.8, 102.9)		0–21		51.9% (–17.4, 179.3)		0–28
Factor 3 (affective symptoms)												
2. Dropped activities/interests		0.98 (0.97, 1.00)		0–21		0.98 (0.94, 1.01)		0–21		0.99 (0.97, 1.01)		0–28
3. Life is empty		1.02 (1.00, 1.03)*		0–7		1.10 (1.04, 1.15)*		0–21		1.04 (1.01, 1.07)*		0–28
4. Often get bored		0.99 (0.97, 1.01)		0–21		1.02 (0.99, 1.05)		0–7		1.01 (0.98, 1.03)		0–28
12. Feel pretty worthless		0.98 (0.96, 1.01)		0–21		1.01 (1.00, 1.03)		0		1.00 (0.97, 1.03)		0–28
14. Situation is hopeless		1.00 (0.99, 1.01)		0		1.03 (0.99, 1.09)		0–21		1.02 (0.98, 1.05)		0–28
15. Others are better off		1.02 (1.00, 1.03)*		0–2		1.05 (1.00, 1.11)		0–14		1.00 (0.96, 1.04)		0–28
Composite score of factor 3c		11.5% (–1.4, 26.1)**		0–2		13.1% (–2.7, 31.4)		0–2		13.0% (–29.5, 81.0)		0–28
aThe model included the following variables: age, sex, number of years of school, BMI, alcohol consumption, regular exercise, creatinine-adjusted urine cotinine level, SBP, triglyceride, daily mean temperature, follow-up time, and day of the week. Estimated effects were expressed as ORs in each individual item and percent change in the composite scores. bPositive symptoms were reverse coded before analysis. ORs > 1 indicate adverse symptoms in every case. cComposite scores were calculated as the loading value-weighted sum of binary scores (0 or 1) of each individual item that contributed to the factor. *p < 0.05. **p < 0.1.												

In our sensitivity analysis, we compared single- and two-pollutant models using moving average lag 2 days for pollutants. Associations of PM_10_ and NO_2_ with depression symptoms were attenuated when the model was adjusted for NO_2_ [17% (95% CI: 5%, 30%) vs. 9% (95% CI: –8%, 30%)] and for PM_10_ [20% (95% CI: 5%, 38%) vs. 11% (95% CI: –10%, 37%)], respectively; however, controlling for other pollutants did not substantially change the risk estimates [see Supplemental Material, Figure S4 (http://dx.doi.org/10.1289/ehp.1104100)]. In an additional sensitivity analysis, we compared estimates in a full model with unweighted or unadjusted estimates. We found that weighted or unweighted estimates were similar, whereas crude effects of PM_10_ and NO_2_ were substantially different from adjusted estimates, although no such difference was evident for O_3_ (see Supplemental Material, Figure S5). The significant confounders in the association with PM_10_ and NO_2_ were age, sex, number of years of school, day of week, and follow-up time.

## Discussion

Our study found that increasing concentrations of PM_10_, NO_2_, and O_3_ were significantly associated with depressive symptoms measured repeatedly among an elderly population in Korea. Individual test items related to emotional symptoms were more likely to be associated with these three pollutants than were items related to somatic or affective symptoms.

Previous studies showed similar results. Welsch (2006) reported that air pollution was a significant predictor of subjective well-being or happiness, and Szyszkowicz et al. (2009b) reported that air pollution from combustion of fossil fuels in motor vehicles was associated with depressive disorders and suicide attempts (Szyszkowicz et al. 2010). Air pollution was associated with headache and migraine symptoms (Szyszkowicz 2008a, 2008b; Szyszkowicz et al. 2009a, 2009c, 2009d). In contrast with previous study results, we found that SO_2_ and CO did not show significant associations with depression symptoms (negative or null associations, and positive or null associations, respectively). For example, Bullinger (1989) reported that increasing SO_2_ concentrations (measured daily at the monitoring sites) were associated with adverse mood and stress. Szyszkowicz (2007a) reported that SO_2_ and CO, as well as NO_2_ and O_3_, were associated with increased emergency department visits due to depression in warm seasons. It is not clear why the results for SO_2_ and CO in our study were not concordant with other studies, although PM_10_, NO_2_, and O_3_ showed similar results. One possible explanation is that oxidant-producing pollutants such as PM_10_, NO_2_, and O_3_ are more likely to affect depressive symptoms than are SO_2_ and CO because oxidative stress is known to be one of the pathogenetic mechanisms of depression (Maes et al. 2011; Wolkowitz et al. 2011). Other plausible explanations for the discrepancies may be different study populations, exposure levels, measurement errors, adjustment of confounding variables, differences in outcome definitions, and sample sizes used in the studies.

Although mechanistic studies linking air pollution to depression are lacking, Block et al. (2004) demonstrated in a study of cell cultures that diesel-exhaust particles activated microglia and induced dopaminergic neurotoxicity by production of proinflammatory factors and reactive oxygen species. Evidence of interdependent relationships between oxidative pathways and neurotransmitters, hormones, and inflammatory mediators involved in the pathogenesis enhance the plausibility of the oxidative stress hypothesis in relation to depression (Ng et al. 2008). Because airborne particles, nitrogen oxides, and ozone are potent oxidants, air pollution may affect depressive moods by inducing oxidative stress.

Studies have shown that estimated effects of air pollution based on single pollutant models may be different from those estimated using multipollutant models (Bell et al. 2007; Rojas-Martinez et al. 2007; Yang et al. 2005). The results of our sensitivity analysis indicated that associations of PM_10,_ and NO_2_ with depression symptoms in two-pollutant models were attenuated, compared with single-pollutant models; however, controlling for other pollutants did not substantially change the risk estimates. Although there was no clear explanation for dependency of PM_10_ with NO_2_ in this study area, we can speculate that NO_2_ from vehicular combustion emissions may be related to traffic particles such as tire wear, road bitumen, sand traction, and pavement aggregates that may increase concentrations of PM_10_ (Kupiainen et al. 2005).

This study examined which items combined were associated with air pollutants. A factor analysis clustered 15 items into three groups (emotional, somatic, and affective symptoms). Responses related to emotional symptoms were increasingly negative as air pollution increased. However, responses related to somatic and affective symptoms were not significantly associated with increases in air pollution.

CVD is also known to affect depressive symptoms (Musselman et al. 1998) and air pollution has been reported to increase the risk of CVD (Bateson and Schwartz 2004; Stieb et al. 2009; Szyszkowicz 2007b). Therefore, the role of air pollution in relation to the cardiovascular system should be considered when evaluating mechanistic pathways of air pollution effects on depressive symptoms. We examined confounding effects of CVD history on the air pollution–depression relationship. Common CVDs such as hypertension, stroke, MI, and hyperlipidemia were examined as covariates in the model. CVD history did not affect or confound the estimated effects of air pollutants; however, hypertension and hyperlipidemia appeared to modify the depression relationship. Participants without hypertension were at a higher risk for depression in association with air pollution exposure than were those with hypertension, although participants with hyperlipidemia appeared to be at increased risk of depression in association with air pollution, especially in relation to O_3_ exposure. However, caution should be used when interpreting the results because we had no clear explanation for the contradictory results between hypertension and hyperlipidemia and limited power to assess differences.

This study has some strengths and limitations. First, since toxicological studies showed that many health effects were delayed (Coffin and Stokinger 1977), we used moving average lag structures of air pollutant concentrations up to 4 weeks. Second, to the best of our knowledge, this is the first study to investigate associations between specific depressive symptoms among the elderly and air pollution after controlling for individual and temporal effects. A big challenge in the present study was the aging of the participants. For example, the elderly participants may have responded incorrectly to questions on the questionnaire, depending on instantaneous memories or health conditions. To validate the instrument to some extent, a test-retest was conducted. The results provided information regarding the reliability of SGDS-K scores over time. Although the number of observations (*n* = 537) was sufficient to estimate effects of air pollution on depression, our findings may not be generalizable to younger populations or those living in different atmospheric conditions. Lag days of air pollution on depression were selected based on the lowest QIC up to 28 lag days. However, there is no clear biological explanation that the selected lags are more appropriate, and a data-driven approach to find the best lag day may be flawed.

## Conclusions

This is the first report to investigate the relationship between depressive symptoms and air pollution in a panel study. Increasing concentrations of PM_10_, NO_2_, and O_3_ were significantly associated with depressive symptoms among an elderly population in Korea. Among depression symptomatology, emotional symptoms were more likely to be associated with the three pollutants evaluated.

## Supplemental Material

(508 KB) PDF
