# Supplementary material for: Air Pollution and Symptoms of Depression in Elderly Adults
Source: Environ Health Perspect. 2012 Apr 18;120(7):1023–8. doi: 10.1289/ehp.1104100 (PMC3404652; doi:10.1289/ehp.1104100)
Supplement: (508 KB) PDF [file ehp.1104100.s001.pdf]

Supplemental Material

Air Pollution and Depressive Symptoms in Elderly Adults

Youn-Hee Lim[1,2], Ho Kim[1], Jin Hee Kim[3], Sanghyuk Bae[4], Hye Yin Park[5], Yun-Chul Hong[*3,6]

[1]Department of Epidemiology and Biostatistics, Graduate School of Public Health, Seoul National University, Seoul, Republic of Korea

[2]Institute of Health and Environment, Graduate School of Public Health, Seoul National University, Seoul, Republic of Korea

[3]Institute of Environmental Medicine, Seoul National University Medical Research Center, Seoul, Republic of Korea

[4]Environmental Health Center, Seoul National University College of Medicine, Seoul, Republic of Korea

[5]Department of Preventive Medicine, Seoul National University College of Medicine, Seoul, Republic of Korea

[6]Institute of Medical Research, Seoul National University Bundang Hospital, Seongnam, Republic of Korea

Table of Contents

Supplemental Material, Table S1 Pearson's correlation coefficients among SGDS-K items, factor loading from a principal factor analysis, and reliability of a test-retest analysis 3

Supplemental Material, Table S2: Pearson correlation coefficients among five pollutants by follow-up times. 4

Supplemental Material, Figure S1: Hourly air pollution concentrations ((a) PM_{10}, (b) SO_2, (c) NO_2, (d) CO, and (e) O_3) and (f) correlation coefficients among pollutants (Pearson correlations coefficients) during the study period (August 2008 ～August 2010) in Seongbuk-Gu, Seoul. Three pollutants (SO_2, NO_2, CO) showed high value of exposure during the rush hour while O3 exposure was peak on mid-afternoon (3 p.m.). Except O_3, four pollutants were highly correlated with each other. 5

Supplemental Material, Figure S2: Percentage change of increasing Korean version of the Geriatric Depression Scale-Short Form (SGDS-K) score per interquartile range (IQR) of SO2 and CO 8

Supplemental Material, Figure S3: Percentage change of increasing Korean version of the Geriatric Depression Scale-Short Form (SGDS-K) score per interquartile range (IQR) of air pollutants (3-year-followup study using repeated measurement (solid circle) vs. cross-sectional study using baseline data (circle)) 9

Supplemental Material, Figure S4: Percentage change of increasing Korean version of the Geriatric Depression Scale-Short Form (SGDS-K) score per interquartile range (IQR) of air pollutants at lag 0-2 in single- and two-pollutant models. 10

Supplemental Material, Figure S5: Percentage change of increasing Korean version of the Geriatric Depression Scale-Short Form (SGDS-K) score per interquartile range (IQR) of air pollutants (Estimated effects derived from fully adjusted/weighted (solid circle) vs. unadjusted (circle) vs. unweighted (triangle) models) 11

Supplemental Material, Table S1 Pearson’s correlation coefficients among SGDS-K items, factor loading from a principal factor analysis, and reliability of a test-retest analysis

Items	Reliability[a]/ Eigen value (% of variance)[e]	Pearson’s correlation coefficients[f]														
		1	2	3	4	5	6	7	8	9	10	11	12	13	14	15
SGDS-K score (1-15)	0.92															
1. Satisfied with life	0.68	1														
2. Dropped activities/interests	0.86	0.24	1													
3. Life is empty	1.00	0.31	0.29	1												
4. Often get bored	0.67	0.37	0.34	0.44	1											
5. In good spirits	0.84	0.4	0.28	0.33	0.41	1										
6. Fear bad things	0.84	0.27	0.16	0.32	0.35	0.32	1									
7. Happy most of the time	0.84	0.49	0.22	0.31	0.39	0.58	0.29	1								
8. Often feel helpless	0.84	0.3	0.14	0.25	0.28	0.27	0.36	0.33	1							
9. Prefer to stay home	0.37	0.3	0.12	0.1	0.23	0.2	0.14	0.28	0.32	1						
10. Problems with memory	0.81	0.07	0.12	0.08	0.12	0.12	0.12	0.08	0.13	0.26	1					
11.Wonderful to be alive	0.84	0.3	0.26	0.39	0.39	0.47	0.3	0.5	0.29	0.08	0.09	1				
12. Feel pretty worthless	0.65	0.27	0.28	0.28	0.33	0.22	0.15	0.23	0.22	0.06	0.1	0.34	1			
13. Full of energy	0.78	0.31	0.29	0.18	0.23	0.2	0.19	0.28	0.2	0.22	0.2	0.25	0.25	1		
14. Situation is hopeless	1.00	0.24	0.34	0.32	0.38	0.28	0.23	0.3	0.31	0.12	0.15	0.31	0.36	0.14	1	
15. Others are better off	0.65	0.33	0.2	0.33	0.41	0.37	0.22	0.35	0.31	0.17	0.14	0.34	0.33	0.19	0.49	1
Factor 1 (Emotional)	4.9 (32%)[e]	0.62[b]	0.10	0.44	0.49	0.73[b]	0.54[b]	0.78[b]	0.49[b]	0.32	-0.10	0.60[b]	0.12	0.20	0.20	0.37
Factor 2 (Somatic)	1.1 (8%)[e]	0.25	0.18	-0.06	0.12	0.04	0.11	0.12	0.35	0.72[c]	0.71[c]	-0.08	0.07	0.49[c]	0.11	0.11
Factor 3 (Affective)	1.3 (9%)[e]	0.16	0.62[d]	0.48[d]	0.50[d]	0.20	0.14	0.13	0.14	-0.12	0.19	0.39	0.69[d]	0.27	0.69 d	0.54 [d]

[a] Reliability coefficients were derived from a test-retest analysis.
Pearson’s correlation coefficient for SGDS-K score (1-15 points) and Kappa score for item-by-item responses (positive or negative) were used.
Factor loading values at the last three rows are clustered into three factors ([b] Factor 1; [c] Factor 2; [d] Factor3)
[e] Eigen value and percent of variance were derived from a factor analysis based on the baseline data (i.e. first visit only data).
[f] Pearson’s correlation coefficients were based on the baseline data (i.e., first visit only data).

Supplemental Material, Table S2: Pearson correlation coefficients among five pollutants by follow-up times.

Air Pollutants	Mean PM_{10} (μg/m3)	Mean SO_2 (ppb)	Mean NO_2 (ppb)	Max CO (10ppm)
3-year repeated measurements				
Mean SO_2 (ppb)	0.69*			
Mean NO2 (ppb)	0.65*	0.60*		
Max CO (10ppm)	0.69*	0.69*	0.69*	
Max O_3 (ppb)	-0.06	-0.18*	-0.15*	-0.30*
1st follow-up:August - December, 2008 (N=383)				
Mean SO_2 (ppb)	0.72*			
Mean NO2 (ppb)	0.75*	0.68*		
Max CO (10ppm)	0.78*	0.78*	0.77*	
Max O_3 (ppb)	-0.32*	-0.49*	-0.39*	-0.49*
2nd follow-up: April - December, 2009 (N=368)				
Mean SO_2 (ppb)	0.67*			
Mean NO2 (ppb)	0.71*	0.56*		
Max CO (10ppm)	0.67*	0.50*	0.65*	
Max O_3 (ppb)	0.38*	0.34*	0.22*	0.26*
3rd follow-up: March - August, 2010 (N=344)				
Mean SO_2 (ppb)	0.57*			
Mean NO2 (ppb)	0.30*	0.40*		
Max CO (10ppm)	0.47*	0.32*	0.70*	
Max O_3 (ppb)	0.16*	0.08	-0.02	-0.15*

* P-value <0.05

Supplemental Material, Figure S1: Hourly air pollution concentrations ((a) PM_{10}, (b) SO_2, (c) NO_2, (d) CO, and (e) O_3) and (f) correlation coefficients among pollutants (Pearson correlations coefficients) during the study period (August 2008 ～August 2010) in Seongbuk-Gu, Seoul. Three pollutants (SO_2, NO_2, CO) showed high value of exposure during the rush hour while O3 exposure was peak in mid-afternoon (3 p.m.). Except O_3, four pollutants were highly correlated with each other.

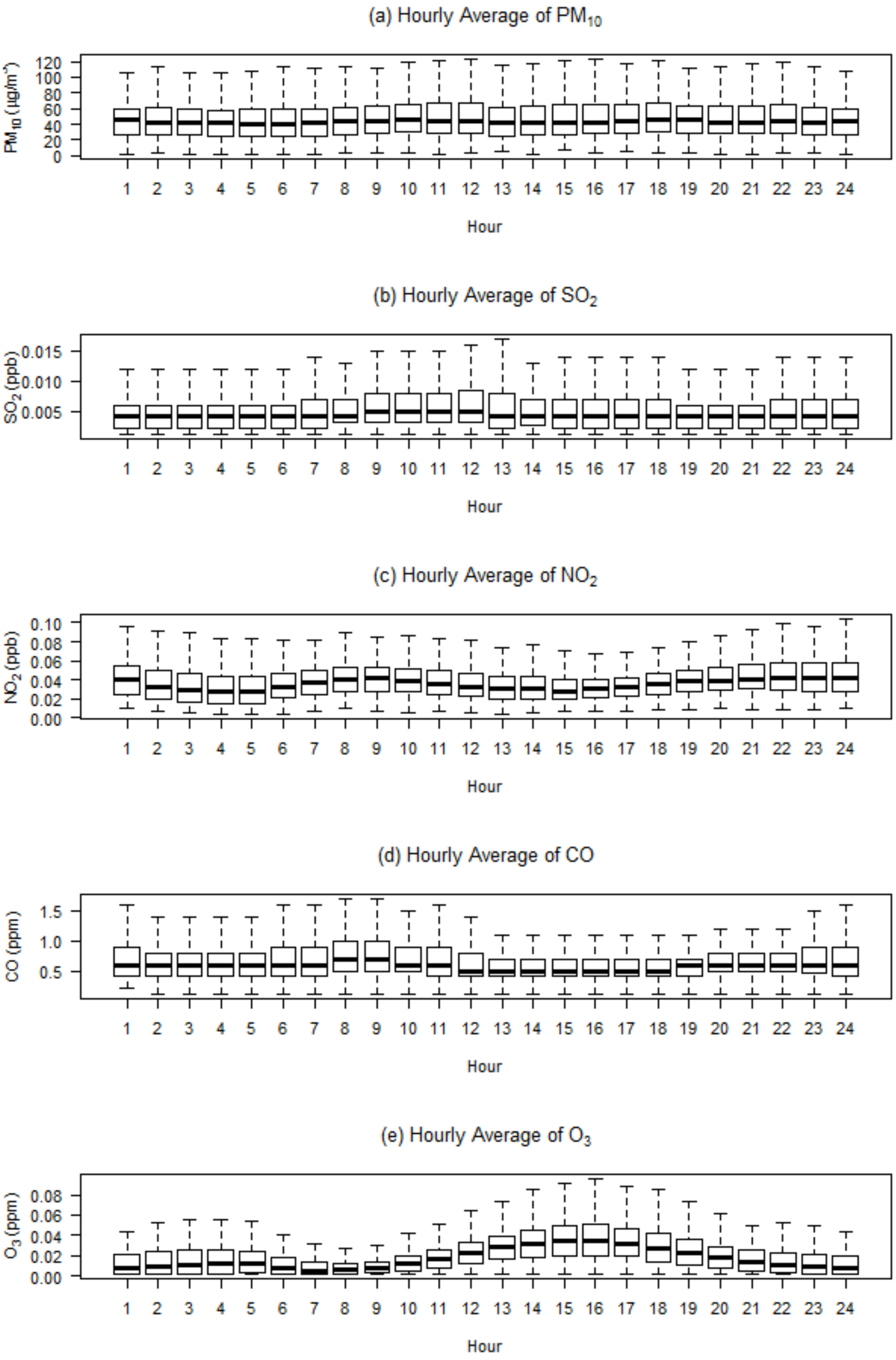

(a) Hourly Average of PM_{10}
PM_{10} (µg/m³)
0 20 40 60 80 100 120
1 2 3 4 5 6 7 8 9 10 11 12 13 14 15 16 17 18 19 20 21 22 23 24
Hour
(b) Hourly Average of SO_2
SO_2 (ppb)
0.005 0.010 0.015
1 2 3 4 5 6 7 8 9 10 11 12 13 14 15 16 17 18 19 20 21 22 23 24
Hour
(c) Hourly Average of NO_2
NO_2 (ppb)
0.00 0.02 0.04 0.06 0.08 0.10
1 2 3 4 5 6 7 8 9 10 11 12 13 14 15 16 17 18 19 20 21 22 23 24
Hour
(d) Hourly Average of CO
CO (ppm)
0.5 1.0 1.5
1 2 3 4 5 6 7 8 9 10 11 12 13 14 15 16 17 18 19 20 21 22 23 24
Hour
(e) Hourly Average of O_3
O_3 (ppm)
0.00 0.02 0.04 0.06 0.08
1 2 3 4 5 6 7 8 9 10 11 12 13 14 15 16 17 18 19 20 21 22 23 24
Hour

(f) Correlation coefficients among pollutants (Pearson correlations coefficients)

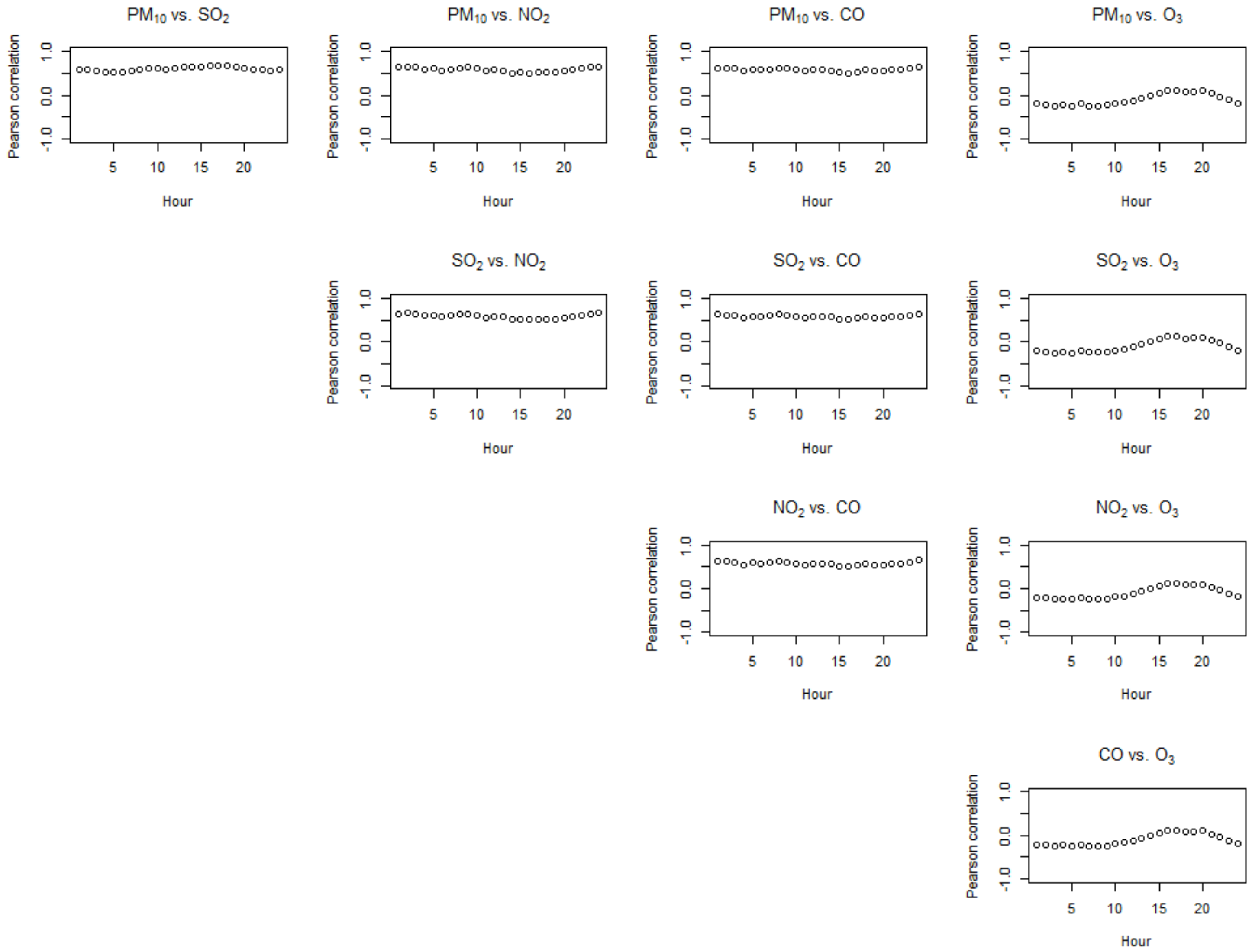

Supplemental Material, Figure S2: Percentage change of increasing Korean version of the Geriatric Depression Scale-Short Form (SGDS-K) score per interquartile range (IQR) of SO2 and CO

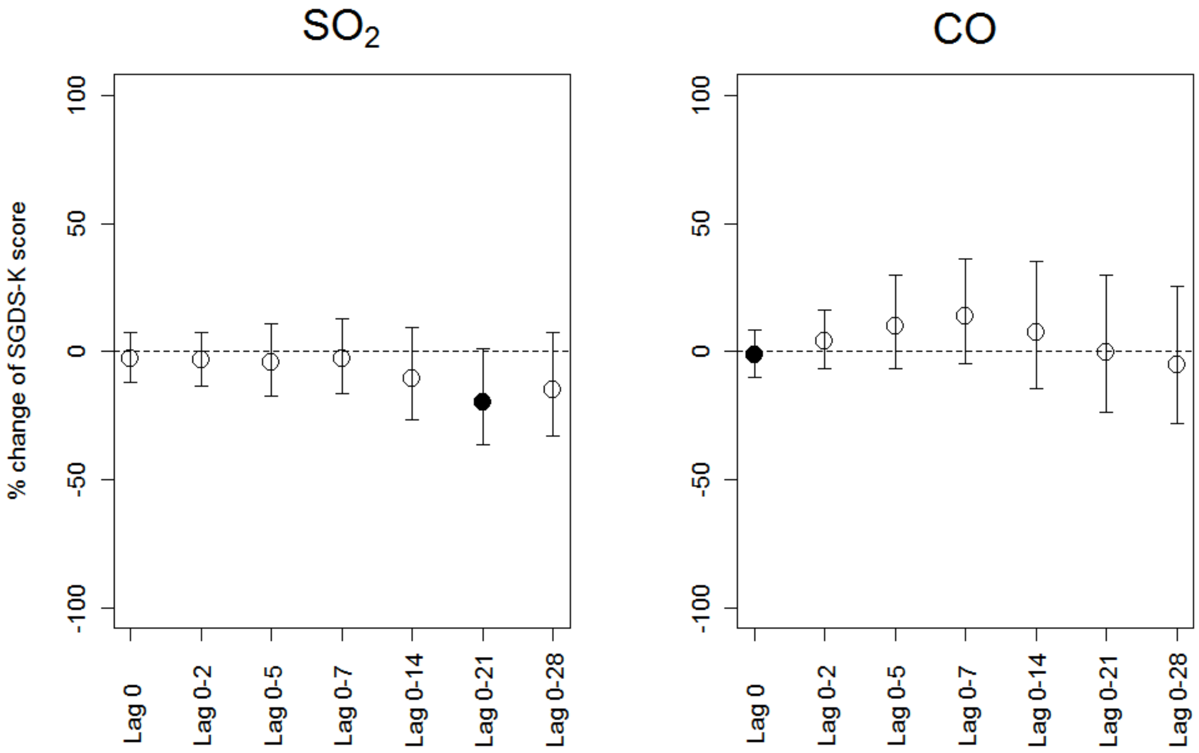

Supplemental Material, Figure S3: Percentage change of increasing Korean version of the Geriatric Depression Scale-Short Form (SGDS-K) score per interquartile range (IQR) of air pollutants (3-year-followup study using repeated measurement (solid circle) vs. cross-sectional study using baseline data (circle))

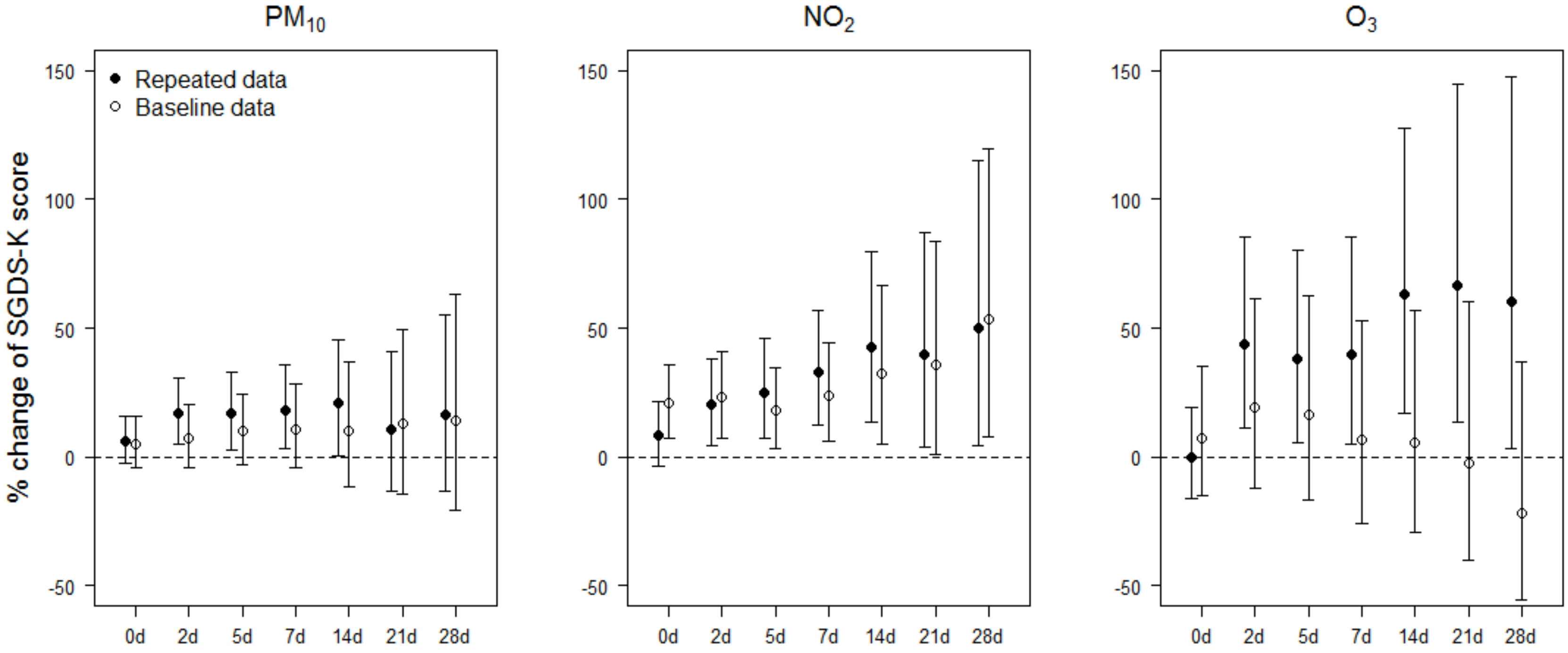

The model for each lag structure included the following variables: age, sex, number of years of schooling, body mass index, alcohol consumption, regular exercise, creatinine-adjusted cotinine level, systolic blood pressure, triglyceride, daily mean temperature, rainfall, follow-up time, and day of the week. A label of '0d' in X-axis means for concurrent exposure to air pollution, '2d' for moving average lag days from concurrent to two previous days, '5d' for 0-5 days of moving average, '7d' for 0-7 days, and '14d' for 0-14 days.

Supplemental Material, Figure S4: Percentage change of increasing Korean version of the Geriatric Depression Scale-Short Form (SGDS-K) score per interquartile range (IQR) of air pollutants at lag 0-2 in single- and two-pollutant models.

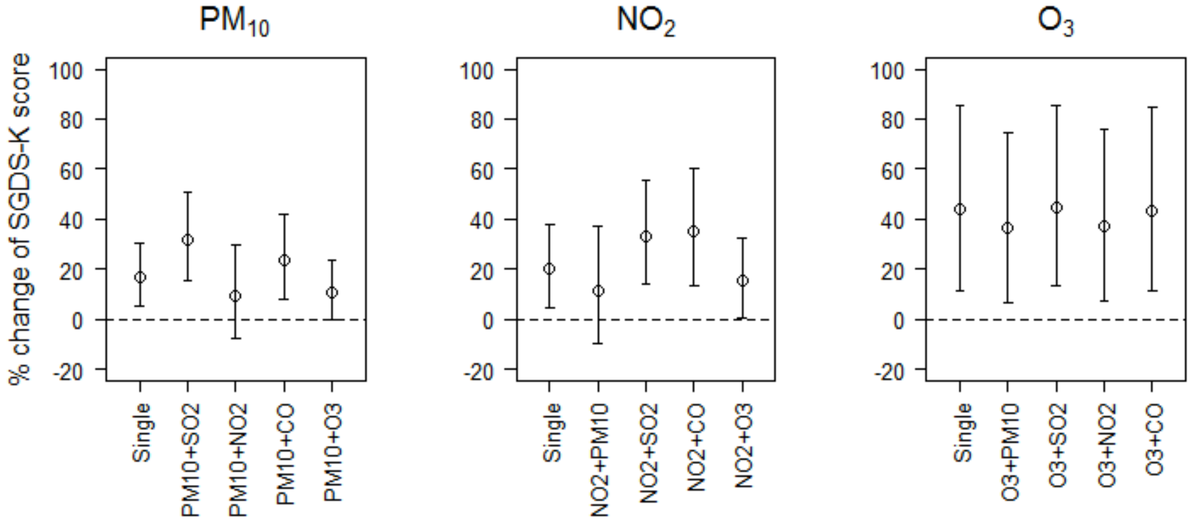

Single- and two- pollutant models included the following variables: age, sex, number of years of schooling, body mass index, alcohol consumption, regular exercise, creatinine-adjusted cotinine level, systolic blood pressure, triglyceride, daily mean temperature, rainfall, follow-up time, and day of the week.

Supplemental Material, Figure S5: Percentage change of increasing Korean version of the Geriatric Depression Scale-Short Form (SGDS-K) score per interquartile range (IQR) of air pollutants (Estimated effects derived from fully adjusted/weighted (solid circle) vs. unadjusted (circle) vs. unweighted (triangle) models)

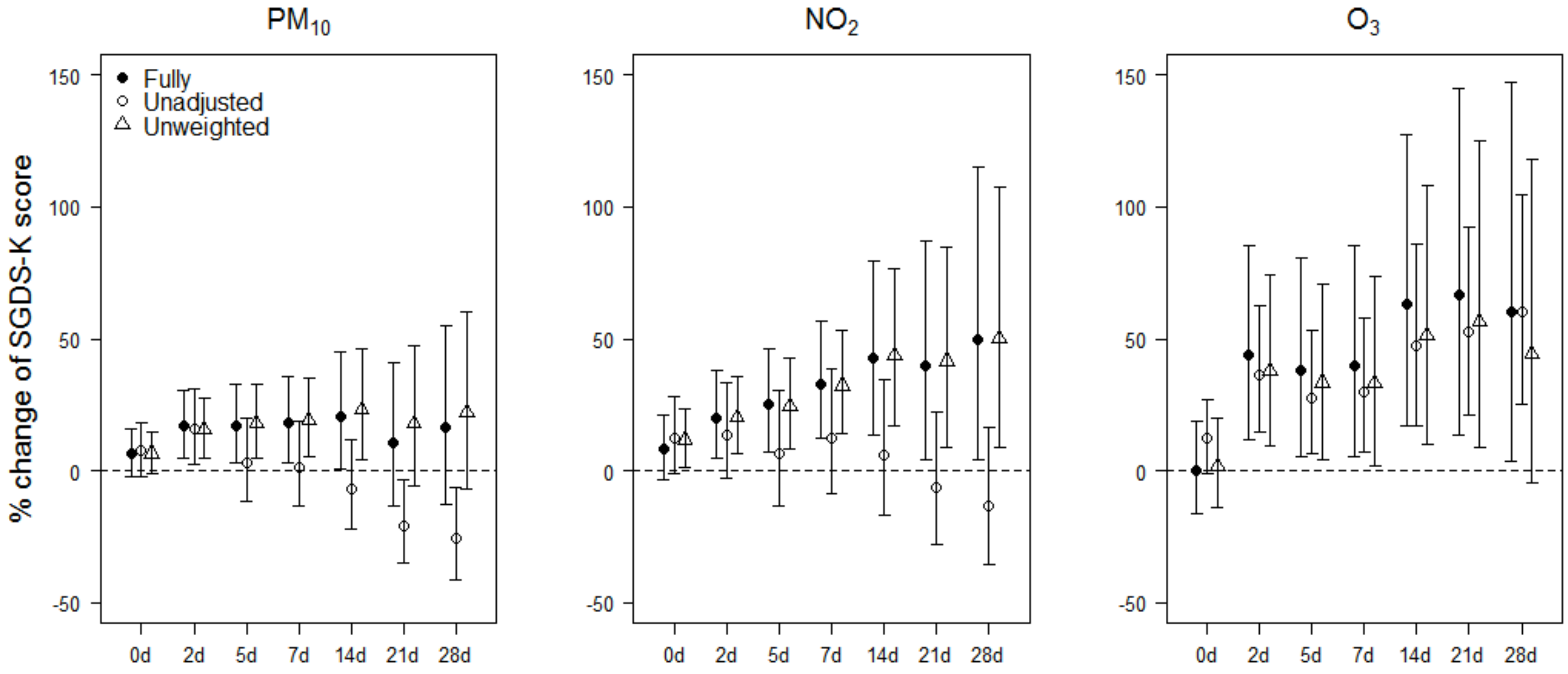

Fully models for each lag structure included the following variables: age, sex, number of years of schooling, body mass index, alcohol consumption, regular exercise, creatinine-adjusted cotinine level, systolic blood pressure, triglyceride, daily mean temperature, rainfall, follow-up time, and day of the week. Unadjusted model includes only response variable and each lag days of air pollution concentrations. Unweighted model includes all variables used in full model except weight of loss of follow-up. A label of '0d' in X-axis means for concurrent exposure to air pollution, '2d' for moving average lag days from concurrent to two previous days, '5d' for 0-5 days of moving average, '7d' for 0-7 days, '14d' for 0-14 days, '21d' for 0-21 days, and '28d' for 0-28 days.
